# Functional integration of LCIB, a component of the algal carbon-concentrating mechanism, enhances carbon assimilation, nitrogen-use efficiency, and biomass in tobacco

**DOI:** 10.3389/fpls.2026.1820295

**Published:** 2026-06-04

**Authors:** Mirna Barsoum, Matthias Buntru, Stefan Schillberg, Greta Nölke

**Affiliations:** 1 Fraunhofer Institute for Molecular Biology and Applied Ecology IME, Aachen, Germany; 2 Institute for Molecular Biotechnology, RWTH Aachen University, Aachen, Germany

**Keywords:** C3 plants, carbon metabolism, carbonic anhydrase activity, CO_2_-concentration mechanism, nitrogen assimilation, photosynthesis, rubisco, transgenic plants

## Abstract

Engineering carbon-concentrating mechanisms (CCMs) into C3 crops is a promising strategy to improve photosynthetic efficiency by increasing the availability of CO_2_ near the active site of ribulose-1,5-bisphosphate carboxylase/oxygenase (RuBisCO), the enzyme responsible for carbon fixation. The biophysical CCM of the green alga *Chlamydomonas reinhardtii* includes stromal limiting CO_2_-inducible protein B (LCIB) protein, which contributes to inorganic carbon retention. Here, we accumulated LCIB in the stroma of tobacco chloroplasts to evaluate its effect on carbon assimilation and plant productivity. LCIB was exclusively localized in the stroma and remained biologically active, leading to a ~3-fold increase in total carbonic anhydrase activity and a lower apparent CO_2_ compensation point (-10%). This resulted in a significantly 12% higher net CO_2_ assimilation rate under ambient conditions, reflecting an increase in photochemical performance including a higher electron transport rate and effective quantum yield of PSII (+14%). Consistent with improved assimilation, LCIB lines accumulated higher levels of soluble sugars and end-of-day starch. Untargeted metabolomics revealed widespread increases in the levels of amino acids and tricarboxylic acid cycle intermediates, suggesting enhanced integration of fixed carbon into nitrogen metabolism. LCIB lines also accumulated twice as much biomass as wild-type counterparts during early development, resulting in up to 19% more fresh weight (FW) and 15.4% more dry weight (DW) at the end of vegetative growth. These lines also maintained higher chlorophyll levels and elevated nitrate reductase activity under limiting nitrogen conditions, and accumulated substantially more shoot (+ 79% FW; + 55% DW) and root (61% FW; 46% DW) biomass while C/N ratios remained similar to wild-type plants. Together, these findings demonstrate that stromal expression of the algal CCM protein LCIB enhances photosynthetic carbon assimilation, supports coordinated carbon–nitrogen metabolism, and improves biomass accumulation in a C3 plant without reconstructing a complete CCM. The targeted enhancement of stromal inorganic carbon retention is therefore a useful incremental strategy to improve C3 photosynthetic performance.

## Introduction

Ribulose-1,5-bisphosphate carboxylase/oxygenase (RuBisCO) catalyzes the incorporation of inorganic carbon into the Calvin–Benson cycle and underpins biomass production in C3 crops, including rice, wheat, soybean, potato, tobacco, and numerous vegetable species. However, RuBisCO has a relatively low catalytic turnover rate and an inherent oxygenase activity that competes with carboxylation. Oxygenation produces the potentially toxic metabolite 2-phosphoglycolate, which must be recycled by photorespiration at an energetic cost and with the net loss of carbon and nitrogen. Under current atmospheric conditions, photorespiration can reduce photosynthetic efficiency by at least 20–30%, particularly when internal CO_2_ concentrations decline due to stomatal limitation or environmental stress (Busch et al., 2013). Improving carbon assimilation in C_3_ crops therefore remains a central objective in plant biology and sustainable agriculture, motivating parallel efforts to enhance RuBisCO activity and elevate CO_2_ levels near the enzyme active site by the introduction of carbon-concentrating mechanisms (CCMs) from heterologous sources (Croce et al., 2024; Gionfriddo et al., 2024; Salesse-Smith et al., 2024).

Multiple photosynthetic lineages have evolved CCMs to mitigate constraints imposed by RuBisCO and limited CO_2_ availability. These include biochemical CCMs in C4 and CAM plants and biophysical CCMs in cyanobacteria and green algae. In microalgae such as *Chlamydomonas reinhardtii*, the CCM elevates CO_2_ levels within specialized pyrenoid structures containing RuBisCO by coordinating the activity of inorganic carbon (Ci) transporters and carbonic anhydrases. Recent structural and systems-level studies have advanced our understanding of pyrenoid assembly, RuBisCO condensation, and stromal Ci retention mechanisms that enable efficient carbon fixation at low CO_2_ concentrations (Barrett et al., 2024; Catherall et al., 2025).

Engineering CCM components into C_3_ plants has emerged as a promising strategy to enhance photosynthetic efficiency and productivity. In addition to the use of transporters and carbonic anhydrases, more recent synthetic biology efforts aim to promote RuBisCO condensation and CCM-like microenvironments within chloroplasts to elevate local CO_2_ concentrations (Adler et al., 2022; Chen et al., 2025). Previous studies have demonstrated the partial functional integration of algal CCM elements into tobacco chloroplasts. Specifically, targeting the Chlamydomonas bicarbonate transporter LCIA and the thylakoid lumen carbonic anhydrase CAH3 to tobacco chloroplasts enhanced photosynthetic performance, carbohydrate accumulation, and biomass production (Nölke et al., 2019). These findings suggest that individual CCM components can function in a C_3_ plant, but successful CCM engineering will probably require not only enhanced Ci uptake but also mechanisms that restrict CO_2_ efflux and stabilize stromal Ci pools.

In parallel, cyanobacterial CCM components have been used to increase Ci levels in the plastids of C3 plants. Early strategies targeted single-subunit cyanobacterial bicarbonate transporters such as BicA and SbtA to the chloroplast inner envelope membrane to increase bicarbonate influx (Rolland et al., 2016; Uehara et al., 2020). More recently, complex cyanobacterial bicarbonate uptake systems, including the Synechococcus ATP-driven BCT1 transporter, have been explored using synthetic biology approaches, although correct sub-compartment localization within the chloroplast and functional activity *in planta* remain major challenges (Rottet et al., 2024). Modeling and carboxysome engineering (Long et al., 2018; Chen et al., 2023) frameworks indicate that combining bicarbonate transporters with microcompartment-based CO_2_ fixation may offer further productivity gains, but the complete functional reconstitution of a cyanobacterial CCM in plant chloroplasts has not been achieved thus far (Nguyen et al., 2024).

Limiting CO_2_-inducible protein B (LCIB) is a key stromal component of the Chlamydomonas CCM. It localizes to a structure surrounding the pyrenoid and is essential for acclimation to low and very low CO_2_ concentrations (Wang and Spalding, 2006, 2014a, b; Yamano et al., 2022). LCIB-deficient mutants exhibit an “air-dier” phenotype, characterized by lethality at low CO_2_ levels but near-wild-type growth at very low CO_2_ levels (Wang and Spalding, 2006; Moroney and Ynalvez, 2007). LCIB functions downstream of the thylakoid lumen carbonic anhydrase CAH3 and contributes to stromal CO_2_ retention. It may facilitate vectorial carbon flux by promoting stromal conversion of CO_2_ to HCO_3_^-^, thereby sustaining the bicarbonate pool that supplies CAH3-mediated CO_2_ regeneration in the pyrenoid. Structural, phylogenetic and heterologous expression studies support a carbonic anhydrase-like activity for LCIB and its proposed role in stromal Ci recapture (Jin et al., 2016; Kasili et al., 2023).

Despite its central role in the algal CCM, it remains unclear whether LCIB can confer measurable benefits in a C3 chloroplast environment that lacks a pyrenoid and dedicated Ci transport architecture. Because LCIB is proposed to act at the level of stromal CO_2_ retention or recapture, it provides a unique opportunity to test whether reducing effective CO_2_ leakage alone can enhance carbon assimilation and metabolic coordination without reconstructing an entire CCM. Here, we expressed Chlamydomonas LCIB in the chloroplast stroma of tobacco and determined its effects on photosynthetic performance, biomass accumulation, and the integration of carbon and nitrogen metabolism under greenhouse conditions at ambient CO_2_ levels. We also evaluated plant performance under low-nitrogen conditions, linking carbon assimilation capacity to nitrogen allocation and use efficiency.

## Materials and methods

### Plasmid DNA, bacteria and plants

The binary vector pTRAkc (Sack et al., 2007) was used to express recombinant LCIB in plants. *Escherichia coli* strain DH5α was used for general cloning. *Agrobacterium tumefaciens* strain GV3101:pMP90RK was used for plant transformation. Leaf discs from 4–5-week-old wild-type tobacco (*Nicotiana tabacum* cv. Petit Havana SR1) plants were transformed by infection with *A. tumefaciens* carrying the recombinant vector (containing the LCIB gene alone or fused to EmGFP). Tobacco plants were cultivated in the greenhouse in DE73 standard soil at 22/20 °C day/night temperature and 70% relative humidity with a 16–h photoperiod. The plants were irrigated with 0.1% (w/v) Ferty 2 Mega (Kammlott, Thüringen, Germany). The seeds were pre-selected on Murashige & Skoog (MS) medium containing kanamycin (0.1 mg mL-1). Four-week-old seedlings were initially transferred to 1.5-L pots in the greenhouse, then 4–5 weeks later into 13-L pots. Unless otherwise indicated, T2 and/or T3 generation plants were used in this study. LCIB transgenic lines, wild-type (WT) and a nonrelated T4 transgenic line (producing the human antibody M12, Schillberg et al., 2019) as a vector control (VC) were also hydroponically grown in the greenhouse under the conditions described above (but the daytime temperature was between 25 and 38 °C, summer time) in low-nitrogen medium (75% less nitrogen than normal MS medium).

### Construction of the bacterial and plant expression cassettes

The *C. reinhardtii* cDNA encoding LCIB (AB168093) was codon optimized for tobacco and synthesized by Genscript (Piscataway, NJ, USA). Correct synthesis was confirmed by sequencing. LCIB was directed to the stroma using the native *C. reinhardtii* target peptide. The LCIB cDNA was also fused to the EmGFP gene (Prasher et al., 1992) to confirm the correct localization of the recombinant protein by confocal microscopy. The LCIB sequence with and without the EmGFP fusion was inserted into pUC18 at the EcoRI and XbaI sites and from there into the plant expression vector pTRA downstream of the constitutive cauliflower mosaic virus double enhanced 35S promoter (Kay et al., 1987), generating the final vectors pTRA-LCIB and pTRA-LCIB-EmGFP.

### Total soluble protein extracts, immunoblot analysis and ELISA

The upper, fully expanded leaves from 4–5-week-old tobacco plants were ground to a fine powder under liquid nitrogen, and total soluble protein (TSP) was extracted as previously described (Nölke et al., 2009) with two volumes of 50 mM Tris-HCl (pH 8) containing 100 mM NaCl, 10 mM dithiothreitol (DTT), 5 mM ethylenediaminetetraaceticacid (EDTA) and 0.1% (v/v) Tween-20. One tablet of protease inhibitor (Roche, Mannheim, Germany) was added per 50 mL of extraction buffer. The extracts were centrifuged (8500xg, 20 min, 4 °C) and the supernatants were used for immunoblot analysis. LCIB was detected using mAb54 (Rasche et al., 2011) diluted 1:5000, which binds to the C-terminal tag54 on the recombinant protein. In parallel, ELISA was used to quantify the amount of recombinantly protein produced in transgenic tobacco lines. For this, TSP extracts were analyzed using an anti-Tag54 antibody, with a purified Tag54-tagged recombinant protein of known concentration used to generate a standard curve. Samples and standards were analyzed under identical conditions, and absorbance was measured at the appropriate wavelength using a microplate reader. Recombinant protein levels were calculated based on the standard curve and expressed as µg recombinant protein per g fresh weight and, where indicated, as a percentage of TSP.

### Carbonic anhydrase activity assay

Carbonic anhydrase activity was measured as described by Wilbur and Anderson (1948) with modifications. Briefly, 100 µL of TSP extract was transferred to 12-mL plastic tubes on ice (two replicates per sample). Carbonic anhydrase from lyophilized bovine erythrocytes (2500 Wilbur Anderson units (WAU) mg-1 protein; Sigma-Aldrich, Steinheim, Germany) was used as a positive control and the TSP extraction buffer as a negative control. We added 3 mL of ice-cold 0.1 M Tris HCl (pH 8.5) then 1 mL of CO_2_-saturated water, and measured the time required for the pH to drop from 8.5 to 6.0. The relative carbonic anhydrase activity was calculated as 10×(Tcontrol/Tsample –1), where Tcontrol = time of the uncatalyzed reaction and Tsample = time of the enzyme-catalyzed reaction. The value was expressed as WAU mg-1 TSP.

### *In vivo* fluorescence microscopy

The expression and subcellular localization of LCIB was determined by affixing a leaf disc (~1 cm^2^) to a microscope slide using distilled water and detecting the LCIB-EmGFP fusion protein using a Leica TCS SP confocal fluorescence microscope (Leica Microsystems, Wetzlar, Germany). The localization of EmGFP (excitation 487 nm, emission 500–570 nm) was compared to chlorophyll (excitation 633 nm, emission 651–714 nm).

### Chloroplast isolation and fractionation

Intact chloroplasts were isolated as previously described (Hardré et al., 2014; Joyard et al., 1982) with modifications. Briefly, we homogenized 17 g of deveined tobacco leaves in 100 mL ice-cold extraction grinding buffer consisting of 0.05 M HEPES-KOH (pH 8.0) with 0.01 M EDTA, 0.33 M mannitol, 0.5 g L-1 bovine serum albumin (BSA), 1 g L-1 sodium ascorbate, 0.001 M MgCl_2_ and one tablet of protease inhibitor cocktail (Roche). The homogenate was centrifuged (1000xg, 10 min, 4 °C) and the pellet was washed with 2 mL washing buffer (0.05 M HEPES-KOH pH 8.0, 0.33 M mannitol). The chloroplasts were purified by centrifugation (4000xg, 15 min, 4 °C) on 40% and 80% Percoll cushions in washing buffer. Intact chloroplasts were collected at the interface between the Percoll cushions. Stromal proteins were separated from the intact chloroplasts as previously described (Olinares et al., 2010) with modifications. Briefly, the chloroplasts were incubated with lysis buffer (62.5 mM Tris-HCl pH 7.5, 2 mM MgCl_2_) for 20 min on ice with mild mechanical disruption. The lysate was then centrifuged (12000xg, 5 min, 4 °C) and the supernatant containing the stromal proteins was collected.

### Gas exchange measurements

Gas exchange was measured in upper, fully expanded leaves from 6–7‐week‐old tobacco plants using an LI-6400 system (Li-Cor, Bad Homburg, Germany) as previously described (Kruger et al., 2006) with the following parameters: photon flux density 1000 µmol m^-2^ s^-1^, chamber temperature 27 °C, flow rate 200 µmol s–1, relative humidity 70%. The photosynthetic rate (A) was measured at different reference CO_2_ concentrations (C_r_) (1000, 600, 400, 300, 200, 150, 100, and 50) (µmol mol-1. A/Ci curve was generated from these data. The Γ value was deduced by regression analysis in the linear range of the A/Ci curve. The apparent photosynthetic rate was measured at varying photon flux densities (50, 100, 200, 400, 600, 800, 900, 1000, 1200 and 1500 µM m-2 s-1) at 1000 ppm CO_2_, and the resulting data were used to generate light response curves.

### Carbon and nitrogen analysis

Total carbon and nitrogen levels in dried, powdered leaf material from LCIB-producing transgenic plants and wild-type controls were measured by gas chromatography using a vario EL III CHNS-O elemental analyzer (Elementar, Hanau, Germany).

### 2D-DIGE and MS

The 2D-DIGE method is described by Horn et al. (2013). MS/MS analysis were carried out as described by Spiegel et al. (2015).

### Quantitation of total soluble sugars and starch in tobacco leaves

Total soluble sugars and starch were measured enzymatically as described by Stitt et al. (1989) and Nölke et al. (2014) with modified preparation steps. For soluble sugars, 40 mg of leaf material from 5–week-old plants at the end of the illumination period was flash frozen in liquid nitrogen and ground in 1 mL pre-chilled 2.5/1/1 (v/v/v) chloroform/methanol/water. The extract was agitated at 4 °C for 10min and centrifuged (16,000xg, 2 min, 4 °C) before mixing 500 µL of the supernatant with 250 µL water and repeating the centrifugation step. The top layer was collected and dried in a speed vacuum concentrator and the glucose, fructose and sucrose concentrations were determined enzymatically.

For starch measurements, 50 mg of leaf material was collected at two different time points: the beginning of the illumination period (7 am, after 1.5 h illumination) and at the end (9 pm). The frozen leaf material was ground in liquid nitrogen and resuspended in 80% (v/v) ethanol. The extract was mixed for 10 min at 80 °C, centrifuged (16,000xg, 20 min, at RT), and the pellet was resuspended in 80% (v/v) ethanol. This step was repeated, and the pellet was resuspended in 50% (v/v) ethanol, followed by mixing at 80 °C and centrifugation as above. The resulting pellet was washed with 90% (v/v) ethanol, resuspended in 400 µL 0.2 M KOH and incubated at 95 °C for 1 h. Finally, the samples were mixed with 70 µL 1 M acetic acid and the starch content was measured enzymatically.

### Leaf metabolites in LCIB transgenic plants

Leaf material was collected 5 h after illumination by picking five well-expanded leaves each from five biological replicates of LCIB transgenic plants and wild-type controls. The leaves were immediately homogenized in liquid nitrogen. All subsequent steps were carried out at Metabolomic Discoveries GmbH (Potsdam, Germany). Derivatization and the analysis of metabolites using a GC-MS 7890A mass spectrometer (Agilent Technologies, Santa Clara, CA, USA) were carried out as previously described (Lisec et al., 2006). Metabolites were identified by comparison to authentic standards in a database (Metabolomic Discoveries GmbH). Liquid chromatography was carried out as previously described (Meiß et al., 2014) and the fractions were analyzed by mass spectrometry using a 6540 QTOF/MS Detector (Agilent Technologies). Metabolite concentrations were normalized to internal standards. Significant concentration changes between samples were confirmed by normal distribution (Shapiro-Wilk test) and variance homogeneity testing (F-test) using appropriate statistical procedures (Student’s t-test, Welch test or Mann-Whitney test). A p-value <0.05 was considered statistically significant.

### Nitrate reductase activity assay

NR activity *in vitro* was measured as described by Luo et al. (2006) with minor modifications. We ground fresh 0.5-g leaf samples in 4 mL extraction buffer (25 mM potassium phosphate pH 7.5, 10 mM cysteine, 1 mM sodium EDTA) followed by centrifugation (4000xg, 15 min, 4 °C). We then mixed 0.4 mL of the supernatant with 1.2 mL of the reaction mixture (0.1 mM potassium phosphate buffer pH 7.5, 0.1 mM KNO_3_) and 0.4 mL 0.25 mM NADH in 0.1 M Tris-HCl (pH 8.0). Controls were prepared for each sample by adding 0.1 M Tris-HCl (pH 8.0) without NADH. The reaction was incubated at 27 °C for 40 min and 200 µL of each sample was then transferred to 96-well plates. The reaction was terminated by adding 50 µL 1% (w/v) sulfanilamide in 3 mM HCl. The nitrite produced in the reaction was measured by adding 0.2% N-(1-naphthyl)ethylenediammonium dichloride followed by colorimetric analysis at 540 nm and was expressed as µM NO^2-^ per gram FW per minute.

## Results

### LCIB is stably expressed in transgenic tobacco and correctly localized to the chloroplast stroma

**Figure 1:**
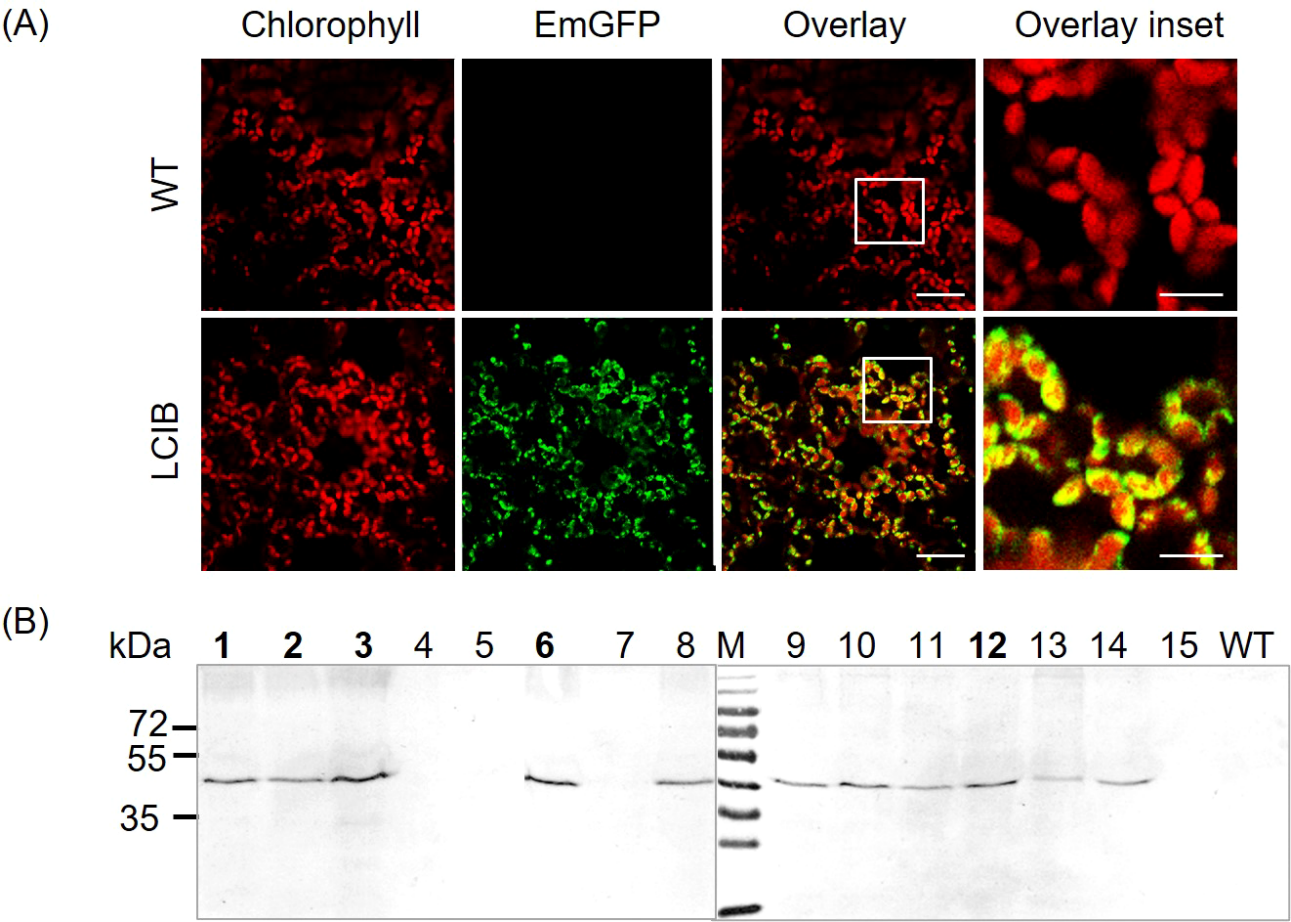
Generation and characterization of transgenic LB lines expressing recombinant LCIB. **(A)** Subcellular localization of recombinant Chlamydomonas LCIB fused to EmGFP. Leaves from tobacco plants transiently expressing LCIB, and wild-type (WT) controls, were imaged by confocal microscopy, showing the localization of LCIB in the stroma of chloroplasts (green fluorescence) and the autofluorescence of the chloroplasts (red). All panels are shown at the same magnification. Magnified insets of the overlay images are shown in the right panels, and the corresponding regions are indicated by white boxes in the overlay images. Scale bars: overlay images = 30 µm; magnified inset = 10 µm. **(B)** Immunoblot analysis of 10 μg total soluble protein (TSP) from crude leaf extracts of 15 independent LB transformants and WT controls. An anti-tag54 antibody was used to detect the ~49-kDa LCIB protein. Lanes 1–15: TSP extracts from representative transgenic lines. WT: TSP extract from WT tobacco leaves. The molecular mass (kDa) of the pre-stained protein marker bands is indicated.

To assess whether LCIB accumulation influences carbon assimilation in a C3 background, we generated transgenic tobacco lines (LB) expressing *C. reinhardtii* LCIB under the control of the double-enhanced constitutive cauliflower mosaic virus (CaMV) 35S promoter and the native *C. reinhardtii* LCIB transit peptide for plastid targeting. Before stable transformation, we transiently expressed an LCIB-GFP fusion protein in tobacco leaves to verify the functionality of the construct. Fluorescence microscopy confirmed the localization of green fluorescent protein (GFP) to the chloroplasts, consistent with stromal targeting (**Figure 1A**).

Molecular screening of 50 independent LB transformants revealed that 56% of the lines expressing lcib mRNA accumulated detectable LCIB protein at levels of 15–45 μg g-1 fresh weight (FW) (**Figure 1B**; **Supplementary Figures 1A, B**). Segregation analysis was carried out in five individual lines producing the highest levels of recombinant LCIB protein (LB-1, 2, 3, 6 and 12) to identify single-locus insertions. Two independent T1 lines (LB-3 and LB-6) showing 3:1 Mendelian segregation, consistent with a putative insertion at a single genetic locus, and high LCIB levels (41 and 33 μg g^-^¹ FW, corresponding to 1.2% and 0.9% of total soluble protein (TSP), respectively) were advanced to homozygosity. Stable expression was maintained through the T3/T4 generations. Analyses were performed across successive homozygous generations to match experimental requirements and material availability; physiological, enzymatic, and biomass analyses were conducted in T2 and T3 plants, while T4 plants were used for evaluation under ambient and low-nitrogen conditions. The consistency of phenotypic and molecular traits across generations indicates stable transgene expression.

**Figure 2:**
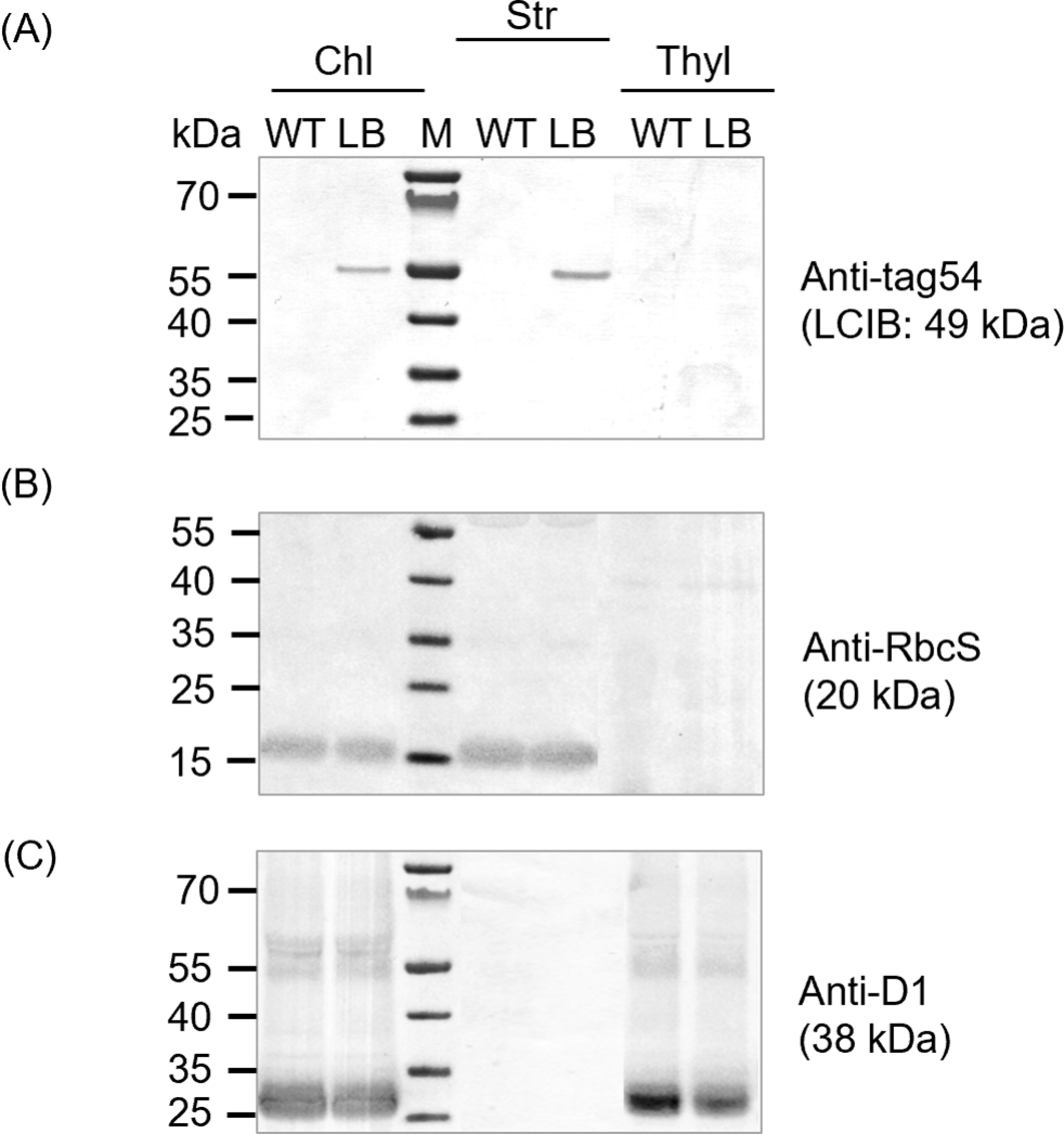
Stromal localization of LCIB in transgenic tobacco confirmed by immunoblot analysis of chloroplast subfractions. Intact chloroplasts were extracted from LB plants (T4) and separated into stromal and thylakoid fractions. Total proteins were also isolated from intact chloroplasts. Immunoblots were probed with antibodies against **(A)** tag54, **(B)** RbcS and **(C)** PsbA/D1 to detect the 49-kDa LCIB, RbcS and PsbA/D1 proteins, respectively. Abbreviations: M, protein marker with the molecular masses (kDa) indicated on the left side; WT, wild-type tobacco plants; LB, transgenic tobacco plants producing recombinant LCIB.

To confirm the subcellular localization of LCIB in stable transformants, intact chloroplasts were isolated from LB-3 plants and wild-type controls, and were fractionated into stromal and thylakoid components by sucrose density gradient centrifugation. Fraction purity was confirmed using stromal (RbcS) and thylakoid membrane (D1) marker proteins. Immunoblot analysis detected LCIB exclusively in the stromal fraction of transgenic plants, with no signal in thylakoid fractions or in any fraction from wild-type plants (**Figure 2**). The absence of LCIB in the membrane fraction enriched for D1 confirmed that recombinant LCIB does not associate with thylakoid membranes in tobacco chloroplasts. Taken together, these results demonstrated the stable expression, heritable integration, and correct stromal localization of LCIB in tobacco chloroplasts, thereby establishing an experimental platform to evaluate its functional impact on carbon assimilation and metabolism.

### LCIB expression increases carbonic anhydrase activity and chlorophyll content

**Figure 3:**
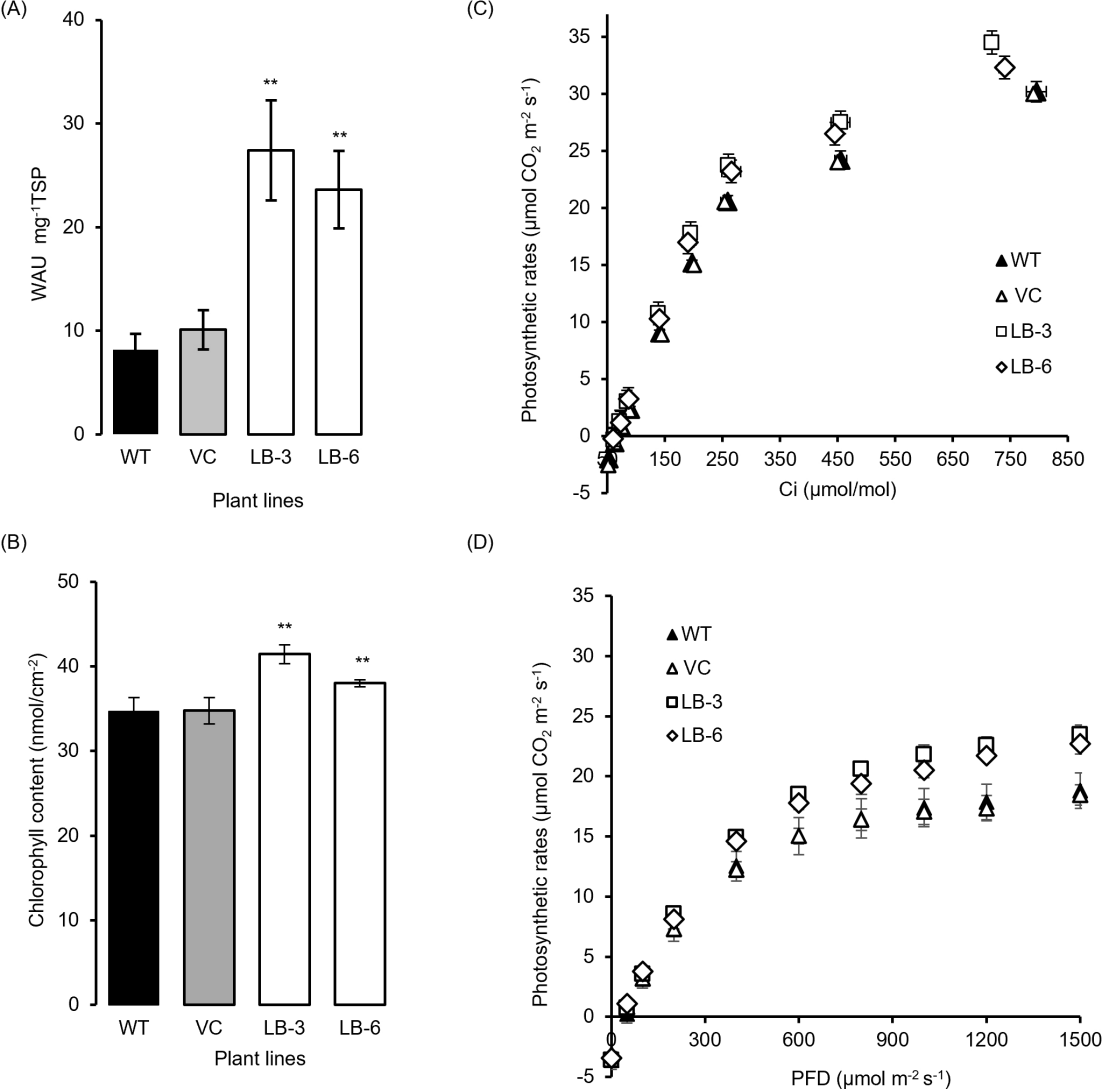
Enzymatic and photosynthetic performance of LB transgenic tobacco plants (T2 and T3) compared to controls. **(A)** Carbonic anhydrase activity in extracts from age-matched, well-expanded leaves obtained from 5-week-old T2 plants. Values are means ± SD (**p < 0.005, n = 5). WAU = Wilbur Anderson units. **(B)** Chlorophyll content of 6–week-old T3 plants. Values are means ± SD (**p < 0.005, n = 5). **(C)** CO_2_ assimilation response curves (A/Ci). Values are means ± SD (n = 3). **(D)** Photosynthetic light response curves. Values are means ± SD (n = 3). WT, wild-type control; VC, vector control (non-related transgenic control); LB-3, transgenic plants producing recombinant LCIB (line 3); LB-6, transgenic plants producing recombinant LCIB (line 6).

To assess whether stromal LCIB expression alters carbonic anhydrase activity in tobacco leaves, we compared total carbonic anhydrase activity in leaf extracts from two independent homozygous transgenic lines (LB-3 and LB-6) with wild-type and vector control plants. Because LCIB has been proposed to exhibit carbonic anhydrase-like activity and to contribute to stromal Ci retention, alterations in total carbonic anhydrase activity would provide functional evidence of its biochemical activity *in planta*. Leaf extracts from LB-3 and LB-6 lines showed significantly higher total carbonic anhydrase activity than wild-type and vector controls. Carbonic anhydrase activity was 3.4-fold higher in LB-3 and 2.9-fold higher in LB-6 (p < 0.0005; **Figure 3A**). The association between LCIB expression and higher carbonic anhydrase activity in leaves is consistent with the proposed function of LCIB.

In addition to altered carbonic anhydrase activity, LCIB-expressing lines contained significantly higher levels of chlorophyll: 19% higher in LB-3 plants and 16% higher in LB-6 plants compared to controls (p < 0.005; **Figure 3B**). Repeated measurements across vegetative development revealed that this increase was maintained throughout the growth period (data not shown), suggesting LCIB has a stable effect on chloroplast-associated traits.

### LCIB expression enhances photosynthetic performance in tobacco

The impact of Chlamydomonas LCIB on photosynthetic performance in the transgenic plants was determined by monitoring gas-exchange parameters in the youngest fully expanded leaves of homozygous LB-3 and LB-6 plants in two independent experiments. Under ambient CO_2_ conditions, the net CO_2_ assimilation rate (A) was significantly higher in LB plants than wild-type and vector controls, with an average increase of ~12% (**Table 1**). A/Ci response curves revealed enhanced carbon assimilation across a range of intercellular CO_2_ concentrations (Ci) in LB plants (**Figure 3C**). Notably, the apparent CO_2_ compensation point was significantly lower (-10%) in both transgenic lines, as determined by regression analysis of the linear portion of the A/Ci curves (**Table 1**), consistent with improved carboxylation efficiency at low Ci.

**Table 1:** Photosynthetic and related performance metrics of LB transgenic tobacco plants in the T2 and T3 generations and wild-type controls.

Photosynthesis parameter	Tobacco lines		Difference
	WT/VC	LB	
A_max_ (µmol m^-2^ s^-1^) (at 400 ppm CO_2_)	20.6 ± 1.1	23.4 ± 1.9 *	+ 12%
Γ (ppm CO_2_)	66.9 ± 2.3	59.9 ± 0.9 *	– 10%
ETR	135 ± 7	150 ± 4.2 *	+ 11%
ΦPSII	0.32 ± 0.027	0.36 ± 0.01 **	+ 14%
F_v_/F_m_	0.83 ± 0.02	0.82 ± 0.01	–

Six independent plants per generation and genotype were analyzed for each measurement in two independent experiments. Values are means ± SD (*p < 0.05, **p < 0.005, n = 24 plants). A significant increase or decrease in the transgenic lines is indicated as a percentage compared to wild type. Amax, apparent CO_2_ assimilation; Γ, apparent CO_2_ compensation point; ETR, electron transport rate; ΦPSII, efficiency of PSII photochemistry Fv/Fm, maximum quantum efficiency of PSII; WT, wild-type control; VC, vector control (non-related transgenic control); LB, transgenic plants producing recombinant LCIB protein (lines 3 and 6).

Light response measurements showed a higher light saturation point in LB plants compared to wild-type and vector controls (**Figure 3D**), suggesting an increased capacity for photosynthesis under conditions of higher irradiance. In contrast, there was no significant difference in the light compensation point, indicating that basal photosynthetic requirements were unchanged. Both the electron transport rate (ETR) and effective PSII quantum yield (ΦPSII) were also significantly higher in LB plants (ETR + 11% and ΦPSII +14%; **Table 1**), consistent with enhanced photochemical efficiency and an increased capacity for carbon assimilation.

Photosynthetic parameters were indistinguishable between wild-type and vector control plants (**Figures 3C, D**), confirming that the observed effects can be attributed to LCIB. Collectively, these results demonstrate that stromal LCIB expression enhances photosynthetic capacity in tobacco under ambient growth conditions.

### LCIB expression enhances growth and biomass accumulation

**Figure 4:**
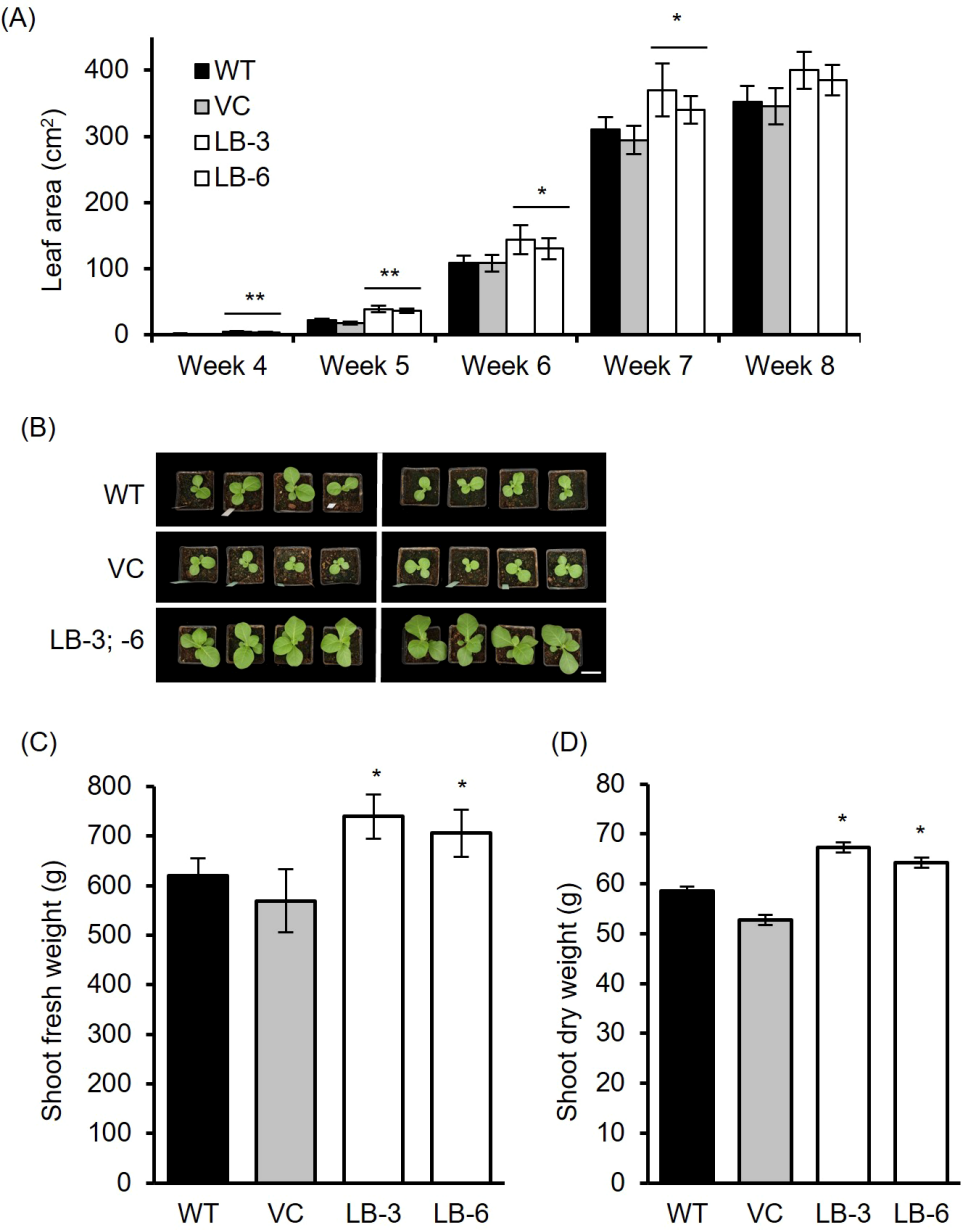
LCIB expression in tobacco leads to an increase in biomass. **(A)** Total leaf area measured weekly (week 4 to week 7, flowering). Values are means ± SD (*p < 0.05, **p < 0.005, n = 10). **(B)** Phenotypes of representative 4-week-old plants. **(C)** Shoot fresh weigh (T3). Values are means ± SD (*p < 0.05, n = 5). **(D)** Shoot dry weight (T3). Values are means ± SD (*p < 0.05; n = 5). WT, wild-type control; VC, vector control (non-related transgenic control); LB-3, transgenic plants producing recombinant LCIB protein (line-3); LB-6, transgenic plants producing recombinant LCIB protein (line-6). Bar = 5 cm.

To determine whether the enhanced photosynthetic performance of LB plants translates into increased growth, we monitored plant development by measuring the leaf area at weekly intervals from the early vegetative stages until flowering (weeks 4–7). Both LB-3 and LB-6 showed accelerated growth and a greater cumulative leaf area than wild-type and vector control plants (**Figures 4A, B**). The growth advantage was evident from early developmental stages, with transgenic plants showing approximately a 100% (**Supplementary Figure 1D**) increase in biomass compared to wild-type, and persisted throughout the vegetative phase. Vector control and wild-type plants grown under identical conditions showed comparable growth dynamics, supporting that the observed phenotype in LB plants was attributable to LCIB expression.

To quantify final biomass accumulation, we measured shoot fresh (FW) and dry weight (DW) at the end of the vegetative stage (week 8; T3 generation). LB-3 plants showed a significant increase in shoot FW (19%) and DW (15.4%) compared to wild-type plants (p < 0.05; **Figures 4C, D**) and the trend was similar in LB-6 plants (p < 0.05). There was no significant difference between vector control and wild-type plants. These findings demonstrate that LCIB expression confers enhanced growth and biomass accumulation under ambient greenhouse conditions.

### Targeted proteomic adjustments in LCIB-expressing plants

To assess whether LCIB expression results in global proteomic remodeling or more targeted changes, we compared leaf samples from LB-3 (T2) and wild-type plants grown under identical conditions by two-dimensional difference gel electrophoresis (2D-DIGE) followed by tandem mass spectrometry (MS/MS) to identify proteins with differences in abundance. Only three proteins exceeded a difference threshold of 1.7-fold (**Supplementary Table 1**). Three were more abundant in LB plants: the RuBisCO large subunit (RbcL), elongation factor P (EF-P), which is required for translational elongation and efficient peptide synthesis and thioredoxin a chloroplast redox protein involved in light-dependent regulation of Calvin–Benson cycle enzymes and maintenance of redox homeostasis. In contrast, a transketolase from the Calvin–Benson cycle that regenerates ribulose-1,5-bisphosphate (RuBP) was less abundant in LB plants. The limited number of changes, involving only four proteins associated with photosynthetic carbon metabolism or protein biosynthesis, suggests that LCIB expression does not induce widespread perturbations but is associated with discrete adjustments in components linked to carbon fixation and translational capacity.

### LCIB expression enhances the accumulation of photosynthetic end products

To determine whether the enhanced photosynthetic performance of LB plants affects carbon allocation, we measured the levels of soluble sugars and starch in the fully expanded leaves of 5–week-old plants. At the end of the light period, we detected higher concentrations of the rapidly metabolized monosaccharides glucose and fructose (1.6-fold increase), as well as the major transport disaccharide sucrose (1.4-fold increase), in LB transgenic plants relative to wild-type controls (**Table 2**). The levels of starch, the principal transient storage form of fixed carbon in leaves, were comparable between LB and wild-type plants in the early morning (8 am), but the LB plants had accumulated ~1.8-fold more starch by the end of the light period (9.30 pm) (**Table 2**). These findings indicate that LCIB expression leads to increased daytime carbon fixation and the accumulation of photosynthetic end products without altering overnight starch mobilization, consistent with enhanced net carbon assimilation.

**Table 2:** Starch and soluble sugar levels in the LB transgenic plants (T2) and wild-type (WT) controls.

Parameter	WT	LB
Glucose	64 ± 9	104 ± 19 *
Fructose	22 ± 6	36 ± 4 *
Sucrose	518 ± 120	725 ± 35 *
Starch	26 ± 2	48 ± 5 *

Soluble sugar levels are presented in µmol m^-2^. Starch levels are expressed as µmol glucose equivalents per gram fresh weight (measured at the end of the day). Values are means ± SD (*p < 0.05, n = 5).

### LCIB expression induces coordinated changes in carbon and nitrogen metabolism

Untargeted metabolic profiling provided a global view of the altered metabolism associated with LCIB expression. Among more than 2000 detected features, 642 metabolites showed significant changes in abundance in LB-3 plants compared to wild-type controls, including 96 annotated compounds (**Supplementary Table 2**).

**Figure 5:**
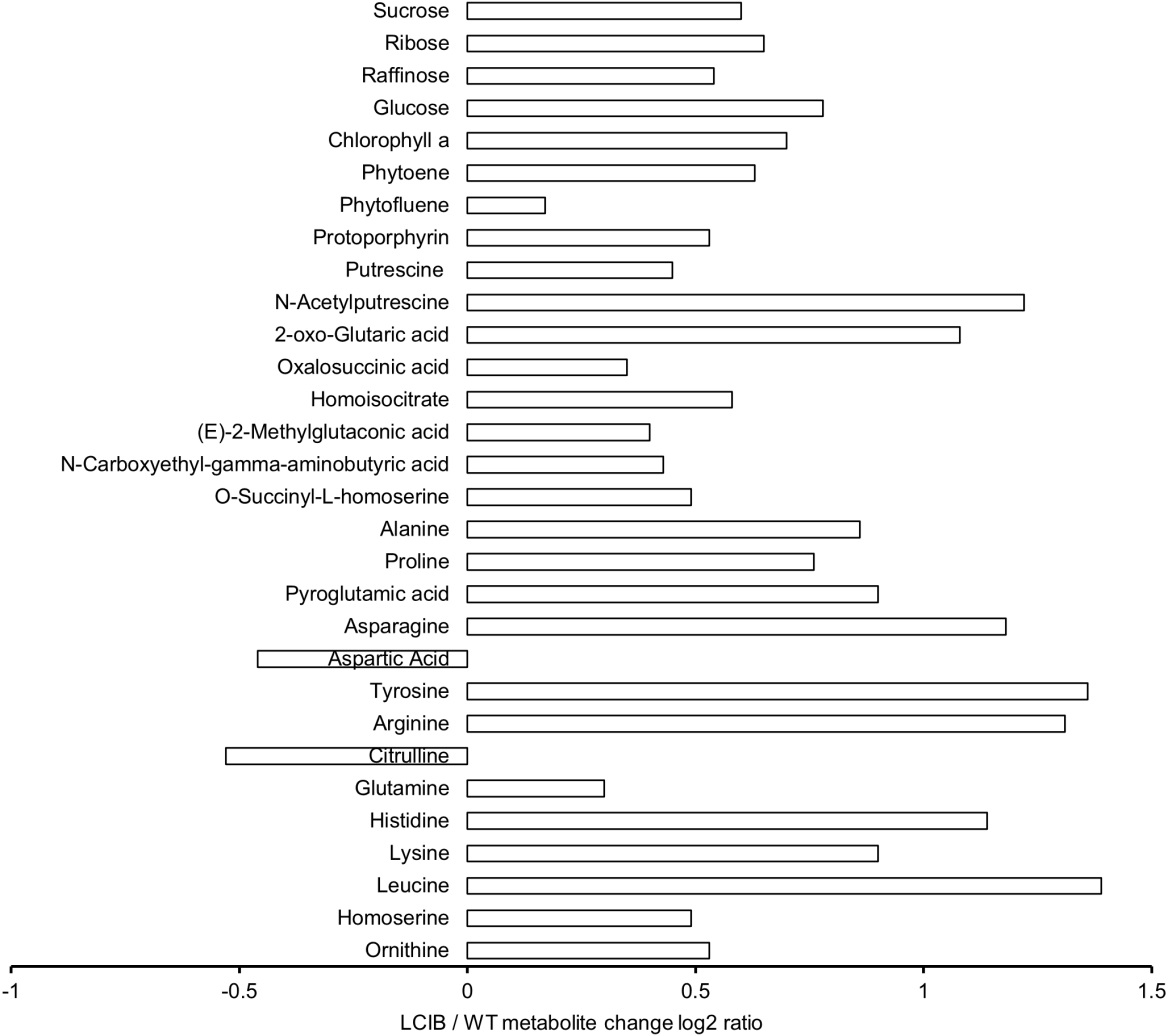
Metabolite levels in the leaves of LCIB-expressing transgenic plants (LB) and wild-type (WT) controls (T2). Samples were collected 5 h after illumination from well-expanded leaves representing n = 5 plants. The fold change values of selected metabolites that significantly (p < 0.05) changed in LB plants compared to WT controls are presented on a log2 scale and are means of five independent lines. The significance of the changes was evaluated using Student’s t-test.

Enhanced carbohydrate pools in LB plants were consistent with the enzymatic sugar assays (**Table 2**). However, metabolic profiling not only confirmed the higher levels of sucrose, glucose and fructose, but also ribose, raffinose and glucopyranose (**Figure 5**; **Supplementary Table 2**). We also observed the differential abundance of amino acids and other nitrogen-associated metabolites in LB plants. Amino acids derived from key carbon-skeleton precursors, such as 2-oxoglutarate (glutamine, arginine and proline), pyruvate (alanine and leucine), and ribose-5-phosphate (histidine), were more abundant in LB plants. In contrast, citrulline and aspartate were less abundant. Metabolites associated with the ornithine-arginine pathway, including arginine, ornithine, and the polyamine precursor putrescine, were significantly elevated in LB plants (**Figure 5**; **Supplementary Table 2**). These changes indicate the more efficient incorporation of fixed carbon into amino acids and other nitrogen-containing metabolites, consistent with enhanced carbon–nitrogen metabolic coupling.

In agreement with the higher spectroscopic chlorophyll readings, chlorophyll a and its precursor protoporphyrin were significantly more abundant in LB plants, and chlorophyll b showed a similar increasing trend. Levels of carotenoid intermediates and precursors, including phytoene and phytofluene, were also higher in LB plants (**Figure 5**; **Supplementary Table 2**). Amino acids linked directly to chlorophyll biosynthesis, such as glutamate and glutamine, were more abundant in LB plants, consistent with enhanced tetrapyrrole metabolism. Intermediates of the tricarboxylic acid (TCA) cycle, including 2-oxoglutarate and oxalosuccinate, were also more abundant in LB plants (**Figure 5**; **Supplementary Table 2**), suggesting the greater availability of carbon skeletons for amino acid biosynthesis. Collectively, our metabolomic data indicate that LCIB expression enhances carbon fixation and promotes the coordinated redistribution of carbon skeletons to primary metabolism, including sugar accumulation, amino acid biosynthesis, and pigment production. An interactive visualization of the comparative biochemical pathway changes in LB and WT plants is presented in **Supplementary Figure 2**.

### LCIB expression enhances biomass and nitrate reductase activity under nitrogen limitation

**Figure 6:**
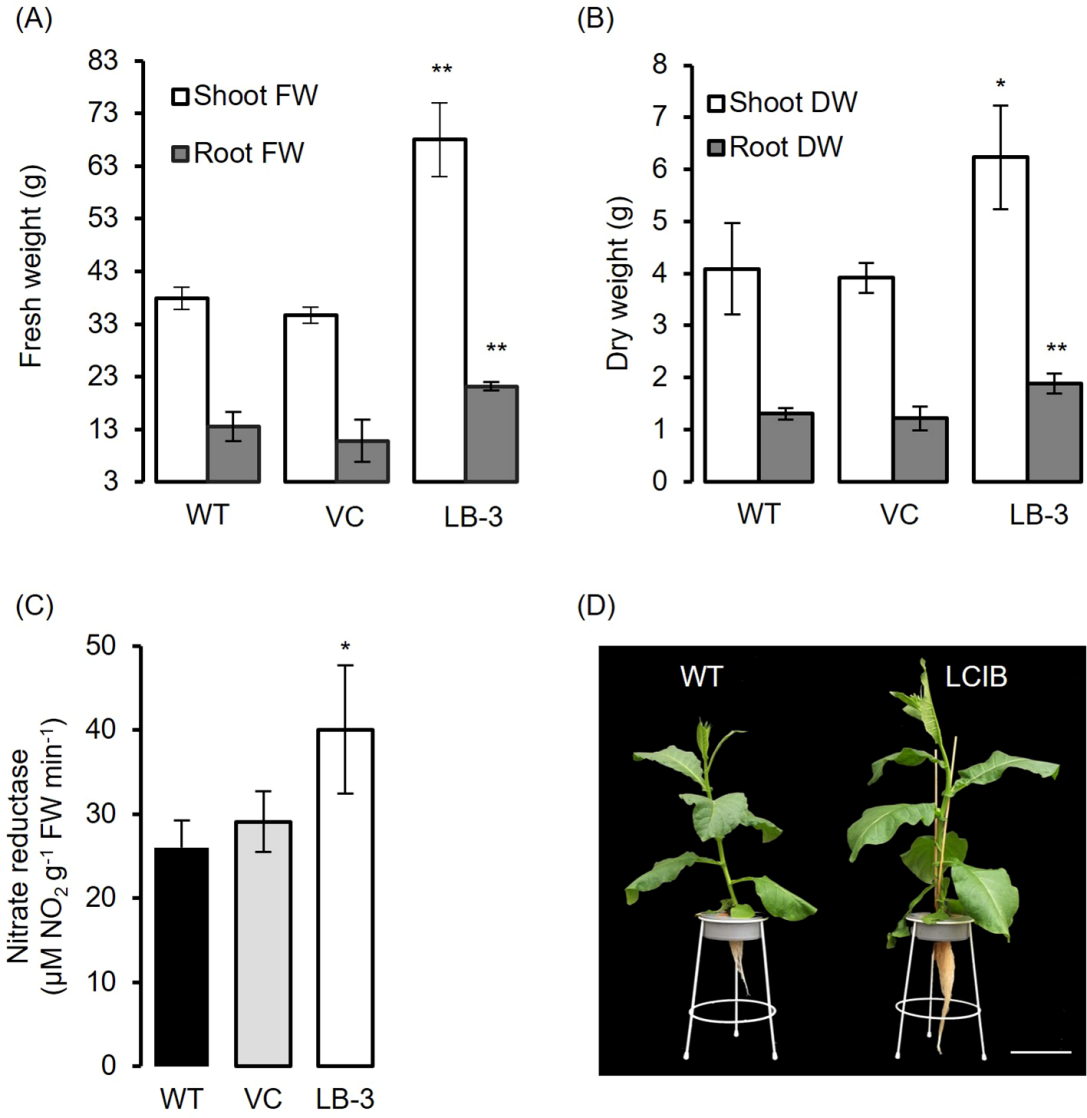
LCIB expression in tobacco plants (T4) growing under nitrogen-limiting conditions (25% normal nitrogen concentration in MS medium) leads to an increase in biomass and nitrate reductase activity. **(A)** Shoot and root fresh weight of hydroponically grown plants under nitrogen-limiting conditions. Values are means ± SD (*p < 0.05, **p < 0.005, n = 10). **(B)** Shoot and root dry weight of plants grown as in **(A)**. Values are means ± SD (*p < 0.05, **p < 0.005, n = 10). **(C)** Nitrate reductase activity of plants grown as in **(A)** expressed as µM NO_2_^–^ per gram fresh weight (FW) per minute. Values are means ± SD (*p < 0.05, **p < 0.005, n = 5). **(D)** Phenotypes of a 7–week-old representative LB transgenic line and WT control growing hydroponically under nitrogen-limiting conditions. Bar = 10 cm.

To assess whether LCIB expression confers an advantage under nitrogen limitation, we grew LB-3 (T4 generation) plants along with wild-type and vector controls in a hydroponic system with a limited nitrogen supply (25% of the standard nitrogen concentration) (**Supplementary Figure 3**). Total carbon and nitrogen levels were comparable between LB and control plants both before and after nitrogen depletion, resulting in similar C/N ratios across genotypes (**Supplementary Table 3**). These results indicate that LCIB expression does not alter overall carbon–nitrogen stoichiometry under nitrogen-limiting conditions. Even so, LB plants exhibited sustained growth advantages under nitrogen limitation. The chlorophyll content was significantly higher in LB plants compared to controls before nitrogen limitation (+14%) and after 1 week under nitrogen-limiting conditions were imposed (+7%), showing that photosynthetic capacity was maintained (**Supplementary Figure 3B**). Two weeks after nitrogen-limiting conditions were imposed, LB plants produced significantly more biomass than controls, with an 79% higher shoot FW and a 55% higher shoot DW (**Figures 6A, D**). Similarly, the root FW was 61% higher and the root DW 46% higher in LB plants (**Figure 6B**). Importantly, the shoot-to-root ratio was unchanged, suggesting coordinated growth of above-ground and below-ground tissues.

To examine whether nitrogen assimilation capacity was affected, we measured nitrate reductase (NR) activity at the same stage. LB plants showed a significant 45% increase in NR activity compared to wild-type controls (**Figure 6C**). In contrast, there was no difference in glutamine synthetase (GS) activity between genotypes (data not shown). No significant differences were observed between wild-type and vector control plants for biomass, chlorophyll content, or NR activity (**Figures 6A–C**), confirming that the enhanced performance under nitrogen limitation can be attributed to LCIB. Together, these findings demonstrate that LB plants maintain higher photosynthetic capacity, biomass accumulation and NR activity under limited nitrogen conditions, supporting their improved physiological performance.

## Discussion

### Stromal LCIB expression enhances carbon assimilation in C_3_ chloroplasts

Engineering CCMs into C3 plants is a strategy to enhance photosynthetic efficiency and yield by increasing CO_2_ availability around the RuBisCO active site (Long et al., 2015; Adler et al., 2022; Fei et al., 2022). Here, we demonstrate that expression of the Chlamydomonas stromal CCM protein LCIB in tobacco enhances carbon assimilation, metabolic coordination, and biomass accumulation under both ambient and nitrogen-limiting conditions.

In Chlamydomonas, LCIB is a soluble stromal protein required for acclimation to low CO_2_ concentrations and it may contribute to stromal Ci retention, potentially by facilitating CO_2_–HCO^_3_-^ interconversion via carbonic anhydrase-like activity in the LCIB/LCIC complex (Wang and Spalding, 2006; 2014a). Direct functional evidence for LCIB carbonic anhydrase activity has recently been strengthened in heterologous systems and carbonic anhydrase-deficient backgrounds, providing a mechanistic basis for LCIB effects *in planta* (Kasili et al., 2023). In tobacco, LCIB localized exclusively to the chloroplast stroma and was associated with a marked increase in total carbonic anhydrase activity and a reduced apparent CO_2_ compensation point. Although whole-leaf carbonic anhydrase assays cannot unambiguously separate intrinsic LCIB catalysis from the indirect effects of endogenous networks, the combination of correct stromal targeting, increased carbonic anhydrase activity, and improved low-Ci photosynthetic behavior is consistent with enhanced Ci availability in the chloroplast.

The literature supports the elevation of carbonic anhydrase capacity as a means to improve C3 photosynthesis and growth. Overexpression of a cytosolic C4 carbonic anhydrase (βCA3) from *Flaveria bidentis* in *Arabidopsis thaliana* increased photosynthetic performance, pigment levels, carbohydrate availability, amino acid abundance, and biomass (Kandoi et al., 2022). Moreover, recent work has highlighted the functional importance of carbonic anhydrase in algal CCMs and Ci handling, reinforcing carbonic anhydrase activity as a biologically meaningful lever in carbon acquisition (Shimamura et al., 2024). The widespread distribution of carbonic anhydrase isoforms across chloroplasts, mitochondria, the cytosol, and the plasma membrane in C3 leaves also highlights the central role of this enzyme in intracellular CO_2_ flux and carbon supply to RuBisCO (Ignatova et al., 2019). Within this framework, our results establish LCIB as a stromal CCM component that improves carbon assimilation without full CCM reconstruction.

LB plants were characterized by higher net CO_2_ assimilation rates and a lower apparent CO_2_ compensation point, consistent with improved photosynthetic performance particularly when Ci is limiting (McGrath and Long, 2014). Such behavior is consistent with the class of “partial CCM effects” predicted by recent mechanistic models and engineering frameworks, in which improvements in Ci retention and local CO_2_ availability can elevate carboxylation efficiency even before full CCM reconstruction is achieved (Price et al., 2011; Lin et al., 2014; Fei et al., 2022). Experimental support for this concept has recently emerged in microalgal systems: co-expression of LCIB and LCIA in *Parachlorella kessleri*-I resulted in increased carbonic anhydrase activity, elevated starch accumulation, and substantially enhanced biomass productivity, consistent with functional augmentation of Ci handling without complete CCM restructuring (Singh et al., 2024). Although performed in an algal background, these findings reinforce the principle that modular enhancement of Ci retention or delivery can shift carbon flux and productivity.

Photosynthesis is constrained by both carboxylation and photochemical energy supply (Farquhar et al., 1980; Long et al., 2004). In several engineered systems, increased carboxylation capacity alone has failed to enhance assimilation when electron transport becomes limiting (Raines, 2011). We observed higher electron transport rates and effective PSII quantum yields in LB plants, together with a higher light saturation point, suggesting that photochemical capacity scaled with and support the enhanced assimilation capacity. This aligns with studies showing that carbon-fixation gains require the coordinated management of electron partitioning and energetic capacity rather than single-node optimization (Hubáček et al., 2024). Notably, thioredoxin abundance was also increased in LB plants. Chloroplast thioredoxins function as central redox regulators that couple photosynthetic electron transport to the activation of Calvin–Benson cycle enzymes and broader primary metabolism. Furthermore, thioredoxin systems act as dynamic regulators of chloroplast metabolism, adjusting redox states in response to fluctuations in electron transport and bridging photosynthesis with metabolic pathways beyond carbon fixation (Nikkanen and Rintamäki, 2019; Cejudo et al., 2021). Elevated thioredoxin levels are therefore consistent with reinforced redox-regulatory capacity supporting enhanced carbon assimilation and coordinated metabolic flux, although the specific contribution of individual thioredoxin isoforms cannot be resolved from the present data. The unchanged light compensation point indicates that the basal respiratory–photosynthetic balance was not fundamentally altered, whereas the higher content of pigment precursors/intermediates and chlorophyll are consistent with sustained or enhanced investment in light harvesting. Low CO_2_ compensation points and high assimilation rates are consistent with a reduced relative photorespiratory flux, although photorespiration was not directly quantified in our study. The greater abundance of chlorophyll precursors and glutamate-derived metabolites supports enhanced tetrapyrrole biosynthesis, providing a mechanistic basis for the higher chlorophyll content of LB plants.

### Redistribution of fixed carbon into storage and nitrogen metabolism

Enhanced carbon assimilation translated into larger pools of soluble carbohydrates and elevated end-of-day starch levels without changes in morning starch levels, consistent with increased daytime carbon gains while preserving overnight remobilization. Similar metabolic outcomes have been observed in systems where photorespiratory loss was reduced (South et al., 2019), supporting the link between improved Ci utilization and carbohydrate accumulation. Our metabolomics data showed that multiple amino acids and TCA cycle intermediates were more abundant in the LB plants, including 2-oxoglutarate–linked compounds, consistent with the provision of more carbon skeletons supporting nitrogen assimilation and amino acid biosynthesis (Araújo et al., 2012). Larger pools of glutamine, glutamate, and related amino acids are markers of efficient nitrogen utilization (Foyer et al., 2003; Masclaux-Daubresse et al., 2010), consistent with coordinated carbon and nitrogen metabolism in LB plants. Proteomic analysis identified only four proteins with marked differences in abundance between LB plants and controls, suggesting LCIB expression did not drive broad proteomic remodeling. The disparity between limited proteomic changes and pronounced metabolomic remodeling is consistent with redistribution of metabolic flux driven by enhanced carbon availability rather than large-scale changes in protein abundance. Interpreted conservatively, this supports a model in which a discrete stromal CCM-derived activity is sufficient to shift photosynthetic and metabolic flux without widespread pleiotropic disruption.

### Improved performance under nitrogen-limiting conditions

Carbon and nitrogen metabolism are tightly interconnected, with nitrate reduction representing a major sink for photosynthetically derived reductants and carbon skeletons (Kaiser and Huber, 2001). As nitrogen assimilation is dependent on the availability of carbon skeletons and reducing power derived from photosynthesis, enhanced carbon fixation can therefore indirectly stimulate nitrogen assimilation (Stitt et al., 2002, Nunes-Nesi et al., 2010). Under nitrogen-limiting conditions, LCIB plants maintained higher chlorophyll levels and more shoot and root biomass compared to controls, but total carbon and nitrogen levels remained similar across genotypes, consistent with maintained stoichiometric balance despite higher productivity. A significant increase in NR activity during nitrogen limitation is consistent with elevated nitrate reduction capacity, a nitrogen-assimilation step tightly linked to photosynthetic reductant supply and carbon status (Kaiser and Brendle-Behnisch, 1991). As no differences in glutamine synthetase (GS) activity were observed between genotypes, changes in ammonium assimilation at this enzymatic level are unlikely under the experimental conditions. The enhanced root biomass during nitrogen limitation may further improve nitrogen foraging, reinforcing whole-plant performance under nutrient stress. Together, these results support the interpretation that LCIB expression improves physiological performance when nitrogen constrains photosynthetic capacity, consistent with the broader objective of improving resource-use efficiency by engineering photosynthesis (Long et al., 2025).

### Implications for incremental CCM engineering

The engineering of algal or cyanobacterial biophysical CCMs into C3 plants is hindered by the need to coordinate Ci transport, compartmentalization, and local CO_2_ management around RuBisCO. Recent roadmaps and reviews emphasize stepwise engineering strategies and highlight progress toward the construction of pyrenoid-like features and the definition of operating principles (Fei et al., 2022; Adler et al., 2022; Catherall et al., 2025). Our results demonstrate that a single stromal CCM-associated component can confer measurable improvements in photosynthetic performance and biomass without reconstructing a complete CCM architecture. By enhancing stromal Ci retention and carbon assimilation capacity, LCIB shifts metabolic flux in a beneficial direction without broad proteomic disruption. These findings support an incremental, component-based approach to CCM engineering, whereby discrete functional modules such as stromal Ci retention factors can be introduced and evaluated sequentially, offering a technically feasible pathway toward the progressive improvement of C_3_ photosynthesis.

## Conclusion

Stromal expression of the Chlamydomonas LCIB protein enhances photosynthetic carbon assimilation, promotes coordinated carbon–nitrogen metabolism, and increases biomass accumulation in tobacco under both optimal and nitrogen-limiting conditions. Improving stromal Ci retention is therefore a viable intermediate strategy that complements ongoing efforts to engineer more comprehensive CCM architectures into C3 crops, thus enhancing their photosynthetic efficiency.

## Data Availability

The datasets presented in this study can be found in online repositories. The names of the repository/repositories and accession number(s) can be found in the article/**Supplementary Material**.
