# Supplementary material for: Functional integration of LCIB, a component of the algal carbon-concentrating mechanism, enhances carbon assimilation, nitrogen-use efficiency, and biomass in tobacco
Source: Front Plant Sci. 2026 Jun 4;17:1820295. doi: 10.3389/fpls.2026.1820295 (PMC13275658; doi:10.3389/fpls.2026.1820295)

Supplementary Material

**Supplementary TABLES**

**Supplementary Table 1.** Proteins showing significant changes in abundance in LB transgenic plants (T2) compared to wild-type (WT) controls as determined by 2D-DIGE and MS/MS. Samples were collected from 8-week-old plants (n = 5, one sample per plant). We analyzed 2952 spots in total (*p < 0.05).

| Identified proteins | Fold change |
| --- | --- |
| Photosynthesis | |
| RuBisCO large chain | 3.25 * |
| Thioredoxin | 1.75 * |
| Transketolase | - 2.02 * |
| Peptide biosynthesis | |
| Elongation factor P | 1.97 * |

**Supplementary Table 2.** Metabolite values in the leaves of T2 generation. Normalised values of the peak areas are listed. WT, wild-type control; WT.sd, standard deviation of five wild-type plants; LB, plants producing LCIB in the stroma; LCIB.sd, standard deviation of five LB transgenic plants; P value, Student’s t-test value.

| Metabolite name | LB | LB.sd | WT | WT.sd | p value |
| --- | --- | --- | --- | --- | --- |
| Sucrose | 0.78687103 | 0.124438 | 0.5178574 | 0.0626566 | 0.0051823 |
| Ribose | 0.00617501 | 0.0018147 | 0.0039436 | 0.0005334 | 0.0491521 |
| Raffinose | 0.00028143 | 4.923E-05 | 0.0001929 | 3.716E-05 | 0.0137069 |
| D-Glucopyranose | 0.00483969 | 0.0011149 | 0.0028172 | 0.0009044 | 0.0143362 |
| N-Acetyl-α-neuraminic acid | 0.04824754 | 0.0132575 | 0.0207529 | 0.0030862 | 0.008407 |
| Lactobionic acid (sugar acid) | 0.02220611 | 0.0102912 | 0.0074045 | 0.0028046 | 0.0300039 |
| L-arabitol (sugar alcohol) | 0.04898467 | 0.0061168 | 0.0407522 | 0.0045021 | 0.0442146 |
| Pro Trp Cys Gly | 0.02498223 | 0.003402 | 0.0159676 | 0.0025386 | 0.0017928 |
| Alanine | 0.00654972 | 0.0023858 | 0.0036128 | 0.0010269 | 0.0488516 |
| Proline | 0.00775151 | 0.0011286 | 0.0045772 | 0.0013172 | 0.0036516 |
| Pyroglutamic acid | 0.01116162 | 0.002377 | 0.0059721 | 0.000781 | 0.0060782 |
| Asparagine | 0.0008981 | 0.0001562 | 0.0003951 | 0.000118 | 0.0005622 |
| Aspartic acid | 1.32549349 | 0.263766 | 1.8214928 | 0.2309833 | 0.0136263 |
| Tyrosine | 0.00505452 | 0.0013315 | 0.0019669 | 0.0004484 | 0.0046801 |
| Ala His Ala | 0.01989025 | 0.0028977 | 0.0118612 | 0.0010471 | 0.0020657 |
| Arginine | 1.55675613 | 0.3241743 | 0.6274282 | 0.16408 | 0.0012943 |
| Asn Tyr Gly | 0.01907023 | 0.0046026 | 0.0100243 | 0.0014879 | 0.0093233 |
| Asp Lys Met | 0.0176115 | 0.0009253 | 0.0135677 | 0.0020424 | 0.0079851 |
| Asp Trp Met | 0.06255539 | 0.0202823 | 0.0311638 | 0.0054143 | 0.0234891 |
| Citrulline | 1.52830359 | 0.4520861 | 2.210389 | 0.4446354 | 0.0428304 |
| Cys Ala Asp | 0.15081815 | 0.0497113 | 0.0860666 | 0.0148354 | 0.0410501 |
| Cys His Asn | 0.29187699 | 0.0846752 | 0.1557151 | 0.032758 | 0.019238 |
| Cys His His | 0.01504777 | 0.0024549 | 0.0095419 | 0.0026116 | 0.0089431 |
| DL-ornithine | 0.02072769 | 0.0031225 | 0.0166869 | 0.0015572 | 0.0420449 |
| Glutamine | 16.7482713 | 1.6287615 | 13.606336 | 1.1915498 | 0.0095298 |
| Gly Glu Pro | 0.06390016 | 0.0040301 | 0.0408102 | 0.0143906 | 0.0205697 |
| His Pro Pro | 0.01764533 | 0.0017229 | 0.0141008 | 0.0010758 | 0.0063975 |
| His Trp Ala | 0.01251101 | 0.0013568 | 0.0102648 | 0.0012804 | 0.0274841 |
| Histidine | 0.40213248 | 0.0848056 | 0.1829193 | 0.0366397 | 0.0024579 |
| Ile Phe His | 0.00865503 | 0.000764 | 0.0069368 | 0.000695 | 0.0059663 |
| Leu Pro | 0.01696779 | 0.0021378 | 0.0110412 | 0.0004654 | 0.0027867 |
| Leucine | 0.01829219 | 0.0066909 | 0.0069681 | 0.0033822 | 0.0152174 |
| Lysine | 0.12519434 | 0.0214981 | 0.0670987 | 0.0111959 | 0.00171 |
| O-Succinyl-L-homoserine | 0.10812916 | 0.0172419 | 0.0771116 | 0.006642 | 0.0124911 |
| Ornithine | 0.04920889 | 0.0112424 | 0.0340735 | 0.0047659 | 0.036218 |
| Ser Arg Trp | 0.0123442 | 0.0019445 | 0.009373 | 0.0007513 | 0.0232446 |
| Ser Asn Ser | 0.00807183 | 0.001791 | 0.0052793 | 0.0002337 | 0.0245338 |
| Trp His Arg | 0.01530543 | 0.0010399 | 0.0124474 | 0.0008937 | 0.0017215 |
| Trp Ile Trp | 0.09110625 | 0.0230652 | 0.0536051 | 0.0181078 | 0.0224036 |
| Trp Trp Val | 0.3391083 | 0.0450067 | 0.2472855 | 0.0254569 | 0.0066211 |
| Tyr Trp Asn | 0.01818313 | 0.004617 | 0.0121229 | 0.0022445 | 0.0398825 |
| Tyr Tyr Ser | 0.07566497 | 0.0139305 | 0.04651 | 0.0086678 | 0.0058786 |
| (E)-2-methylglutaconic acid | 0.06353597 | 0.0069628 | 0.0482284 | 0.0057527 | 0.0056676 |
| 2,6-dimethyl-hexadecanoic acid | 0.00078867 | 0.000458 | 0.0093148 | 0.0029584 | 0.0026549 |
| 2-Hydroxyadipic acid | 0.15508542 | 0.0271873 | 0.1057628 | 0.0279018 | 0.0221302 |
| 3-Methyl-pentadecanoic acid | 0.01326727 | 0.0025223 | 0.022413 | 0.0019382 | 0.0002669 |
| 4,7,10,13,16,19-Docosahexaynoic acid | 0.00170484 | 0.0008742 | 0.0005165 | 0.0001699 | 0.0370223 |
| 4,7,10,13-Eicosatetraenoic acid | 0.62684755 | 0.0362398 | 0.4214135 | 0.0923129 | 0.0051306 |
| 4-Amino-4-deoxychorismic acid | 1.41211994 | 0.159862 | 1.1501028 | 0.1832975 | 0.0431272 |
| 5,14,15-Trihydroxy-6,8,10,12-eicosatetraenoic acid | 0.12561229 | 0.0042991 | 0.0924076 | 0.0175874 | 0.0117765 |
| 6,8-Dihydroxy-octanoic acid | 0.02575174 | 0.0049036 | 0.0163087 | 0.0021036 | 0.0091546 |
| Decanoic acid | 0.00168609 | 0.0007779 | 0.0040774 | 0.0007383 | 0.0010805 |
| 2-Oxo-glutaric acid | 0.01446682 | 0.0040606 | 0.0068497 | 0.0023577 | 0.0097768 |
| Dodecanoic acid | 0.00036213 | 0.0001051 | 0.0010572 | 0.0004333 | 0.021064 |
| Putrescine | 0.22191939 | 0.0222136 | 0.1622178 | 0.0134178 | 0.0016134 |
| 2,5-Dihydroxy-benzoic acid | 0.00414774 | 0.0020558 | 0.0099997 | 0.001848 | 0.0015218 |
| Galactonic acid-1,4-lactone | 0.01495631 | 0.004348 | 0.0083783 | 0.0024225 | 0.024175 |
| Phytosphingosine | 0.0749952 | 0.0038039 | 0.0592346 | 0.0102685 | 0.0230017 |
| *N*-Acetylputrescine | 0.22127166 | 0.0423691 | 0.0950036 | 0.0252316 | 0.0009216 |
| Oxalosuccinic acid | 0.06043685 | 0.0058251 | 0.0473381 | 0.0059404 | 0.0078456 |
| Phenethylamine | 0.00709794 | 0.0010359 | 0.0050405 | 0.0005301 | 0.0076067 |
| Acetoacetic acid | 0.40664304 | 0.1247284 | 0.2041295 | 0.0433993 | 0.0189292 |
| 1,2-Epoxy-3,4-butanediol 4-methanesulfonate | 0.02856509 | 0.0068442 | 0.0191441 | 0.002713 | 0.033597 |
| Neoabietal | 0.50092702 | 0.0384211 | 0.3267668 | 0.0853503 | 0.0070191 |
| 3-Methylindole | 1.82973923 | 0.263637 | 1.4925402 | 0.0929645 | 0.0431079 |
| *n*-Heptadecane | 0.00154304 | 0.0007048 | 0.0032435 | 0.0011533 | 0.0275764 |
| D-Pantothenic acid | 0.00080808 | 0.0001181 | 0.0005165 | 0.0001567 | 0.011654 |
| Dihydrosphingosine | 0.00616197 | 0.0001155 | 0.0044903 | 0.000897 | 0.0135152 |
| Arbutin | 0.0102844 | 0.0021389 | 0.0074843 | 0.0003299 | 0.0420158 |
| Flavin adenine dinucleotide (FAD) | 0.02034945 | 0.001731 | 0.0152196 | 0.0025463 | 0.0073149 |
| GPEtnNMe (18:1(9Z)/18:1(9Z))[U] | 0.59214087 | 0.0796751 | 0.4461674 | 0.0644157 | 0.0136281 |
| GPIns (12:0/13:0) | 0.07502924 | 0.0056697 | 0.0614681 | 0.0066177 | 0.0086306 |
| Hesperidin | 0.01549719 | 0.0057202 | 0.0070731 | 0.0006611 | 0.0295867 |
| Homoisocitrate | 0.03815528 | 0.0051203 | 0.0255905 | 0.0065692 | 0.0106045 |
| Indoleacrylic acid | 0.52979102 | 0.0374318 | 0.4127448 | 0.0814004 | 0.0287049 |
| Met-Arg-Phe-Ala acetate salt | 0.20196235 | 0.030329 | 0.1451572 | 0.021177 | 0.0105726 |
| *N*-Acetyl-7-O-acetylneuraminic acid | 0.04861324 | 0.0066164 | 0.0368303 | 0.0020199 | 0.01384 |
| Methyl 8-gingerol | 0.29636555 | 0.0179114 | 0.2035364 | 0.0334454 | 0.001459 |
| PGJ2 | 0.0561177 | 0.0024478 | 0.0373535 | 0.0026399 | 2.805E-06 |
| Stearamide | 0.00041019 | 2.575E-05 | 0.0002941 | 8.603E-05 | 0.0366383 |
| Tuberonic acid | 0.10091631 | 0.0079282 | 0.0655794 | 0.0142497 | 0.0025482 |
| 15-Oxo-ETE | 0.2314209 | 0.01985 | 0.183591 | 0.017602 | 0.003894 |
| Chlorophyll *a* | 0.01568675 | 0.0038044 | 0.0096323 | 0.0017738 | 0.0195582 |
| Phytoene | 0.00469529 | 0.0012112 | 0.0023051 | 0.000717 | 0.0077493 |
| Phytofluene | 0.0080538 | 0.0011259 | 0.0059309 | 0.0010261 | 0.0144617 |
| Protoporphyrin | 0.02054562 | 0.0026474 | 0.0142536 | 0.0028963 | 0.0072259 |
| Fucoxanthinol | 3.42548658 | 0.2055449 | 2.9695152 | 0.3120166 | 0.029712 |
| Methyl jasmonate | 0.1098586 | 0.00853 | 0.091663 | 0.006347 | 0.005852 |
| Queuine | 0.06715679 | 0.0113193 | 0.084359 | 0.0096911 | 0.0331794 |

**Supplementary Table 3.** Total carbon (C), nitrogen (N), and C:N ratio in LB transgenic plants relative to wild-type controls under nitrogen-limited hydroponic conditions. Elemental composition was determined by CHNS-O elemental analysis in 7-week-old plants grown hydroponically with 75% reduced nitrogen supply. Values represent mean ± standard deviation (n = 4).

| Parameter | LB vs WT (% change) |
| --- | --- |
| Carbon (C%) | + 2% |
| Nitrogen (N%) | + 0.3% |
| Carbon / Nitrogen ratio (C / N) | + 0.5% |

**Supplementary Figure 1.** Generation and molecular characterization of LB transgenic plants expressing LCIB. **(A)** PCR amplification of genomic DNA from LB transgenic tobacco plants (T_0_) using gene-specific primers. M, 1-kb DNA ladder; 1–12, Check PCR of 12 T_0_ transgenic plants expressing LCIB using *lcib* gene-specific primers; PC, PCR positive controls (PCR amplification of pTRA-LCIB using the same *lcib* gene-specific primers); WT, PCR amplification of genomic DNA extracted from wild-type tobacco plants using the same *lcib* gene-specific primers. **(B)** Immunoblot analysis of total soluble protein (TSP) from crude leaf extracts of T_0_ transgenic tobacco plants and a wild-type (WT) controls. M, protein markers with molecular masses (kDa) indicated on the left; 1–12, detection of 49-kDa LCIB protein in 12 T_0_ transgenic plants using an anti-tag54 antibody. **(C)** Immunoblot analysis of total soluble protein (TSP) from crude leaf extracts of T1 transgenic tobacco plants and a wild-type (WT) controls. M, protein markers as described above; 1–7, detection of 49-kDa LCIB protein in lines LB-3, LB-6 and LB-1 using am anti-tag54 antibody. **(D)** Phenotypes of representative 3–week-old plants. WT, wild-type control; VC, vector control (non-related transgenic control producing the monoclonal antibody M12); LB, plants producing LCIB in the chloroplast stroma.


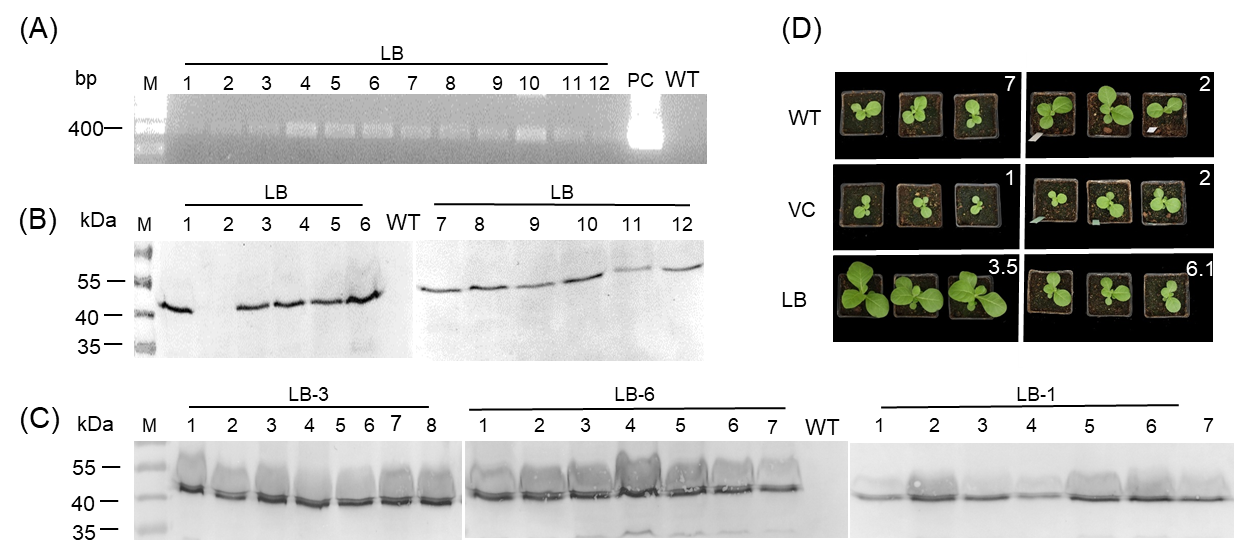


**Supplementary Figure 2.** Visualisation of metabolic pathways of LB line *vs* wildtype using interactive pathway explorer iPath (http://pathways.embl.de/iPath2.cgi). Fold changes (LB/WT) are displayed in color code, while the p-value changes of metabolites in size code.


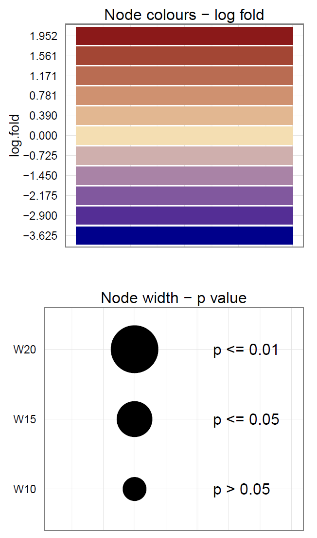

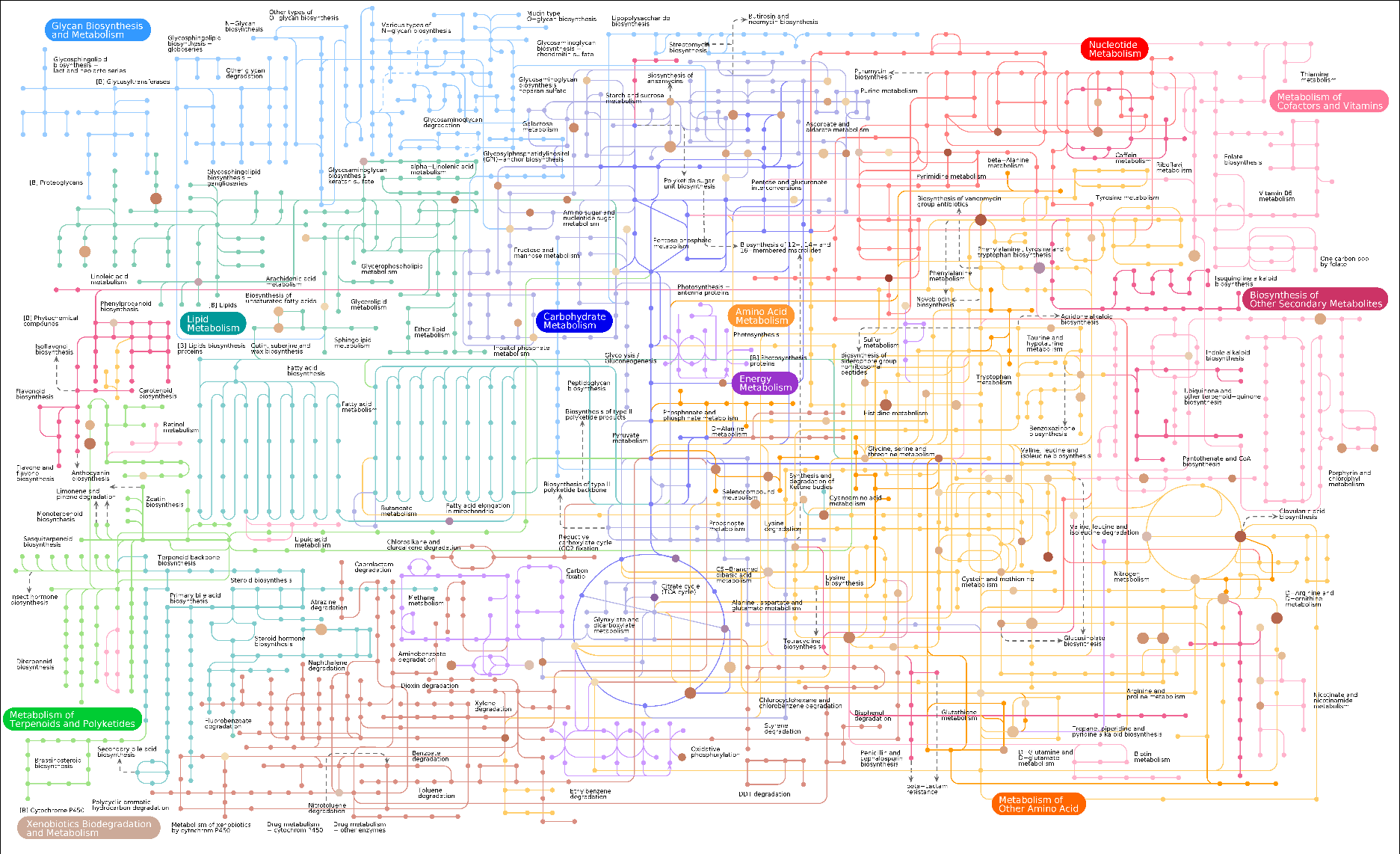


**Supplementary Figure 3.** Representative growth performance of LCIB transgenic plants compared with wild-type controls under nitrogen- depleted hydroponic conditions (A).

Plants were cultivated hydroponically with 75% reduced nitrogen supply for two weeks. Photographs show developmental progression from weeks 1–3 (seedling stage), weeks 4–5 (vegetative development), and weeks 6–7 (late vegetative growth and biomass accumulation). (B) Chlorophyll content of 4–week-old (before treatment) and 7–week-old LB and WT plants. Values are means ± SD (**p < 0.005, n = 5).

(A)


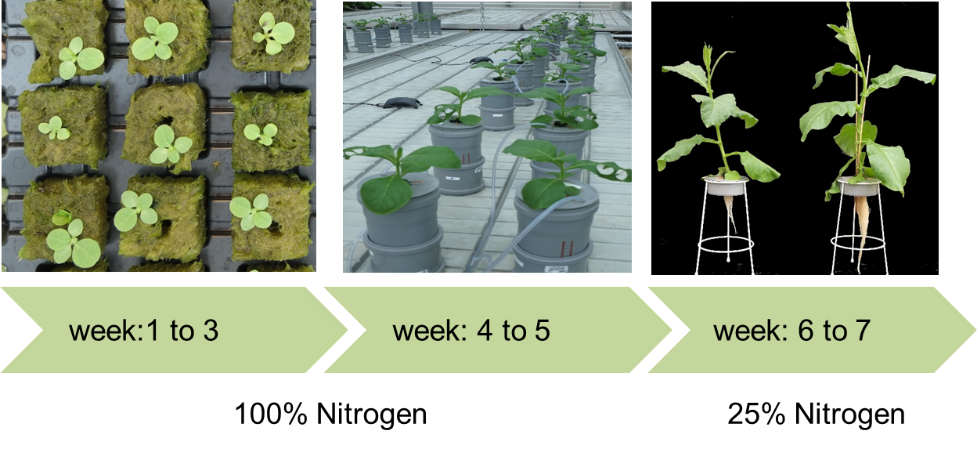


(B)


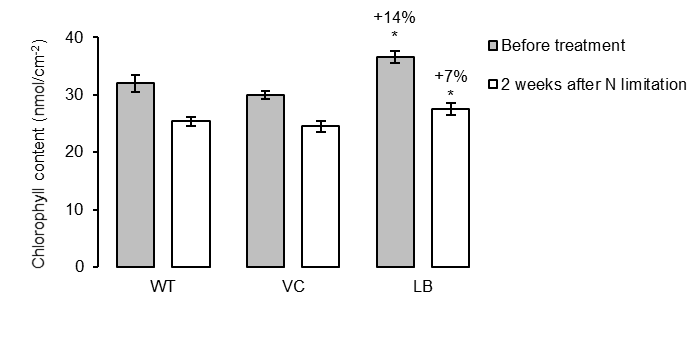

Supplement: Supplementary file 1 [file Table1.docx]
